# *JAM3* methylation status as a biomarker for diagnosis of preneoplastic and neoplastic lesions of the cervix

**DOI:** 10.18632/oncotarget.6250

**Published:** 2015-10-27

**Authors:** Aijun Yin, Qing Zhang, Xiangnan Kong, Lin Jia, Ziyan Yang, Lihua Meng, Li Li, Xiao Wang, Yunbo Qiao, Nan Lu, Qifeng Yang, Keng Shen, Beihua Kong

**Affiliations:** ^1^ Department of Obstetrics and Gynecology, Qilu Hospital, Shandong University, Jinan, P.R. China; ^2^ Laboratory Medicine Center, Qilu Hospital of Shandong University, Qingdao, P.R. China; ^3^ Institute of Pathology and Pathophysiology, Shandong University School of Medicine, Jinan, P.R. China; ^4^ Institute of Diagnostics, Shandong University School of Medicine, Jinan, P.R. China; ^5^ Department of Breast Surgery, Qilu Hospital, Shandong University, Jinan, P.R. China; ^6^ Pathology Tissue Bank, Qilu Hospital, Shandong University, Jinan, P.R. China; ^7^ Department of Obstetrics and Gynecology, Peking Union Medical College Hospital, Chinese Academy of Medical Sciences and Peking Union Medical College, Beijing, P.R. China

**Keywords:** JAM3, cervical neoplasia, methylation, triage, complementary

## Abstract

DNA methylation is clinically relevant to important tumorigenic mechanisms. This study evaluated the methylation status of candidate genes in cervical neoplasia and determined their diagnostic performance in clinical practice. Cervical cancer and normal cervix tissue was used to select the top 5 discriminating loci among 27 loci in 4 genes (*CCNA1, CADM1, DAPK1, JAM3*), and one locus of *JAM3* (region M4) was identified and confirmed with 267 and 224 cervical scrapings from 2 independent colposcopy referral studies. For patients with atypical squamous cells of unknown significance and those with low-grade squamous intraepithelial lesion, with *JAM3*-M4 compared to a triage marker of hrHPV testing, the specificity for cervical intraepithelial neoplasia 3 CIN3 and cancer cases (CIN3+) / no neoplasia and CIN1 (CIN1−) was significantly increased, from 21.88 to 81.82 and 15.38 to 85.18, respectively. The corresponding positive predictive value (PPV) was increased from 26.47 to 57.14 and 18.52 to 63.64, respectively. For hrHPV-positive patients, compared to a triage marker of cytology testing, *JAM3*-M4 showed increased specificity and PPV, from 30.67 to 87.65 and 38.82 to 82.14, respectively. We assessed whether *JAM3*-M4 could distinguish productive from transforming CIN2; the coincidence rate of *JAM3*-M4 and P16 was as high as 60.5%.

## INTRODUCTION

Cervical cancer is a leading cause of cancer deaths in women worldwide, with an estimated 528,000 new cases and 266,000 deaths annually; 85% occur in developing countries [1, 2]. As a developing country, China has a high incidence of cervical cancer, 7.5/100,000 women, and mortality 3.4/100,000 women, especially in rural areas because of lack of proper screening [3]. The incidence of cervical cancer has reached 81/100,000 people in some areas in China [4]. By 2030, cervical cancer is expected to be responsible for the death of 474,000 women annually, with more than 95% of these deaths anticipated to occur in low- and middle-income countries [5]. Thus, establishing a proper screening strategy is of great importance to reduce the cancer burden in China.

The most widely used screening methods for cervical cancer are the cytology-based Pap smear and high-risk human papillomavirus (hrHPV) testing. However, both methods have drawbacks. In one study, of more than 60,000 women, cytology-based Pap smear screening did not detect almost half of the cases of cervical intraepithelial neoplasia 2 (CIN2), CIN3 and cancer (CIN2+) and only 20% of the women with an abnormal Pap smear had histologically confirmed CIN2+ [6]. Moreover, cytology testing is often subjective for a great number of professional cytologists. HrHPV testing of cervical scrapings can improve the sensitivity of cervical screening [7, 8]; however, the lifetime risk of hrHPV infection is estimated to be about 80% [9] and the screening hrHPV test cannot discriminate between infections that would transform into cancer and transient infections. Such a less specific screening test may lead to a substantially heavy burden on health care resources, such as unnecessary referral to colposcopy. To avoid missed diagnoses and over-diagnoses, other triage and/or complementary biomarkers that are molecularly based and not morphology based are urgently needed.

DNA hypermethylation of the promoter and 5′ region of tumor suppressor genes is an epigenetic modification that may be involved in the early phase of carcinogenesis, including cervical carcinogenesis [10–13]. Epigenetic changes occur during each of stage of cervical cancer [14], and various genes are silenced by promoter methylation at distinct stages in the transformation process [15, 16]. The accumulation of epigenetic alterations in the host genome promote the progression to invasive cervical cancer [17]. The most appropriate screening biomarkers of DNA methylation in cervical carcinogenesis appear during the progression to high-grade dysplasia. A series of studies [10, 14, 18, 19] reviewed the epigenetic alterations in premalignant and malignant lesions of the cervix but were heterogenous and results were inconsistent for most genes. Methylation frequencies for the same gene vary widely among studies because of the different loci chosen [20], different genetic backgrounds of various populations [21, 22], specific features of assay protocols, or other unidentified factors.

In the present study, we aimed to identify highly distinguishable DNA methylation loci of genes that may be clinically practical as biomarkers of cervical cancer, especially in geographic locations where quality-controlled cytology testing is absent and a follow-up strategy for HPV-positive women is not defined [23]. Previously reported methylation status in cervical neoplasia identified cyclin A1 (*CCNA1*) [24–26], cell adhesion molecule 1 (*CADM1*) [20, 26–28], death-associated protein kinase 1 (*DAPK1*) [25, 26, 29] and junctional adhesion molecule 3 (*JAM3*) [13] as the most discriminating and stable biomarkers. From microarray data from The Cancer Genome Atlas and a review of the literature to identify the most discriminating loci, we chose various loci of these 4 genes for in-depth investigation by methylation-specific PCR (MSP) of cervical cancer and normal cervical tissue. The markers were further evaluated and confirmed with cervical scrapings from two colposcopy referral studies including 267 and 224 cervical scrapings, respectively (Predictors 1 [P1] and Predictors 2 [P2]), and the most promising marker was evaluated to identify its potential as a triage or complementary marker in hrHPV or cytology testing. Finally, the discriminating marker was validated by pyrosequencing and assessed for potential to triage the controversial CIN2.

## RESULTS

### Sample characteristics

Clinicopathological data for tissue specimens are in Table 1. Clinicopathological data and available hrHPV and cytology results for cervical scrapings are in Table 2.

**Table 1:** Clinicopathological characteristics of the tissue specimen

	No.
Total number	43
Age (Mean ± SD)	45.75 ± 10.21
FIGO	
IA	9
IB	11
IIA	6
IIB	4
IIIA	5
IIIB	6
IV	2
Pathological type	
Squamous carcinoma	35
Adenocarcinoma	7
Others	1

**Table 2:** Clinicopathological data of patients with scrapings and the relative available hrHPV testing and cytology testing results

	Predictors 1					Predictors 2				
	Normal No.	CIN1 No.	CIN2 No.	CIN3 No.	Cancer No.	Normal No.	CIN1 No.	CIN2 No.	CIN3 No.	Cancer No.
Total number	53	59	72	63	20	70	49	51	33	14
Age (Mean ± SD)	44.35 ± 10.39	41.88 ± 8.78	38.31 ± 10.42	40.39 ± 8.49	50.29 ± 11.86	41.84 ± 10.05	40.20 ± 6.99	38.39 ± 8.01	39.61 ± 7.74	46.38 ± 8.73
HrHPV										
Positive	39	41	62	44	9	55	41	41	24	7
Negative	6	10	1	1	0	5	1	0	1	0
Cytology										
NILM	10	16	10	3	1	21	19	9	5	2
ASCUS	16	19	25	8	3	30	22	20	8	2
LSIL	18	9	12	6	1	8	6	7	6	1
ASC-H	3	2	5	14	1	2	2	3	4	3
HSIL	3	1	6	9	2	3	0	6	8	1

### MSP of normal cervical and cervical cancer tissue

**Figure 1:**
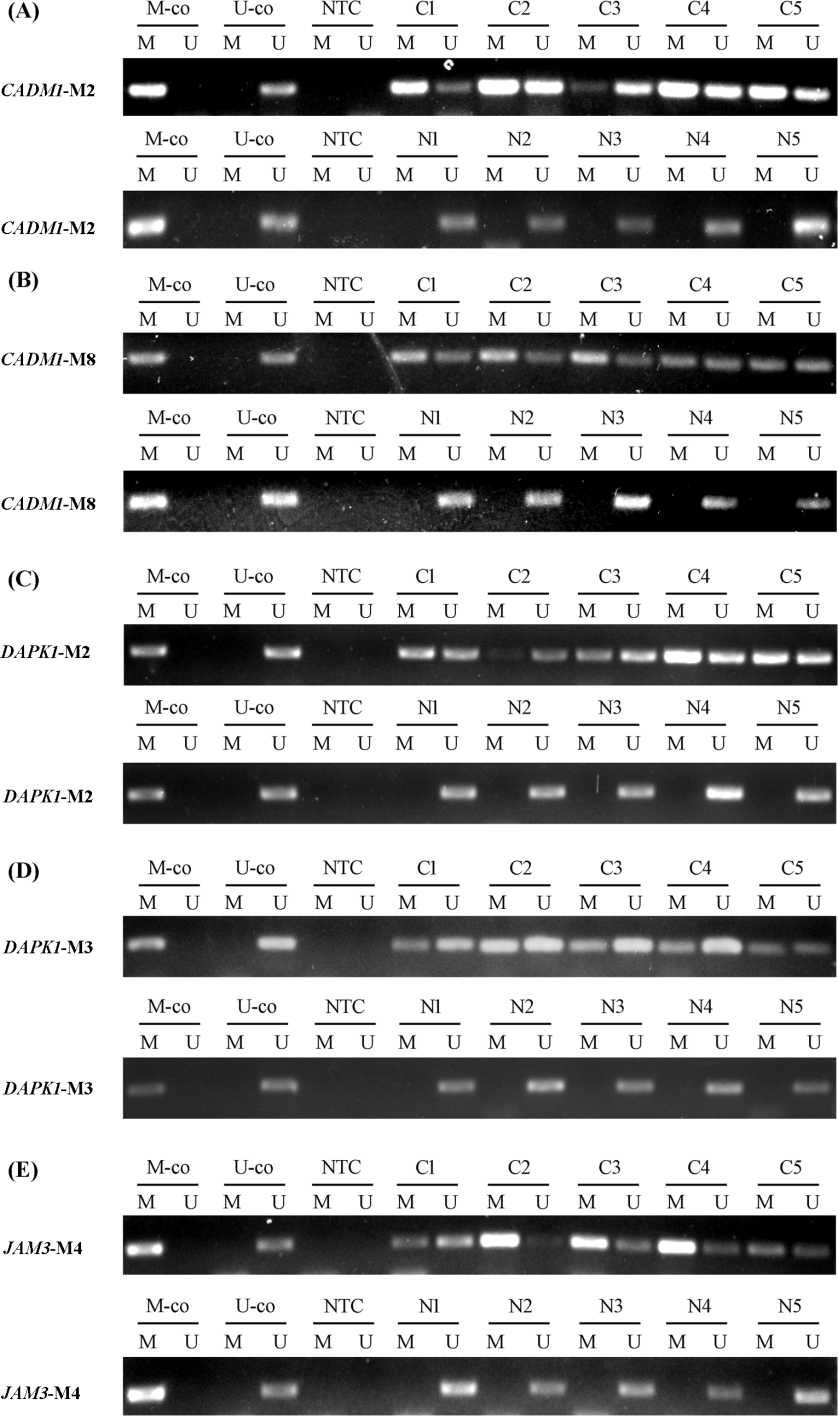
Methylation status of candidate genes in representative examples (**A**) *CADM1*-M2. (**B**) *CADM1*-M8. (**C**) *DAPK1*-M2. (**D**) *DAPK1*-M3. (**E**) *JAM3*-M4. Abbreviations: C: cervical cancer. N: normal cervical tissue. M-co: bisulfite-converted methylated DNA. U-co: bisulfite-converted unmethylated DNA. NTC: no-template control. M: methylated-specific primer sets; U: unmethylated-specific primer sets.

MSP analysis of specimens from 27 patients with normal cervical tissue and 43 with cancer tissue is in Table 3. Promising markers were chosen on the basis of *P* value and comparison of methylation frequency. Liquid-based cytology specimens were the most frequent specimens used for cervical cancer screening and triage. The specific loci for each gene were labeled M1, M2, etc. The first 5 gene loci (*CADM1*-M2, *CADM1*-M8, *DAPK1*-M2, *DAPK1*-M3, and *JAM3*-M4) selected from the initial 27 markers were further evaluated by quantitative MSP (QMSP) in cervical scrapings (Figure 1). The methylation frequency for these loci was significantly higher in cervical cancer than normal cervical tissue (Table 3).

**Table 3:** Methylation frequency in tissue specimen obtained from patients with normal cervix or cervical cancer

Gene-locus	Cancer	Normal	*P*^a^
*CADM1*-M2	28/43	6/27	< 0.001
*CADM1*-M8	32/43	0/27	< 0.001
*DAPK1*-M2	30/43	3/27	< 0.001
*DAPK1*-M3	33/43	2/27	< 0.001
*JAM3*-M4	37/43	2/27	< 0.001

a *P* value was calculated by chi-square test. If groups were too small, Fisher's exact test was applied

### QMSP of cervical scrapings as a screening biomarker

**Figure 2:**
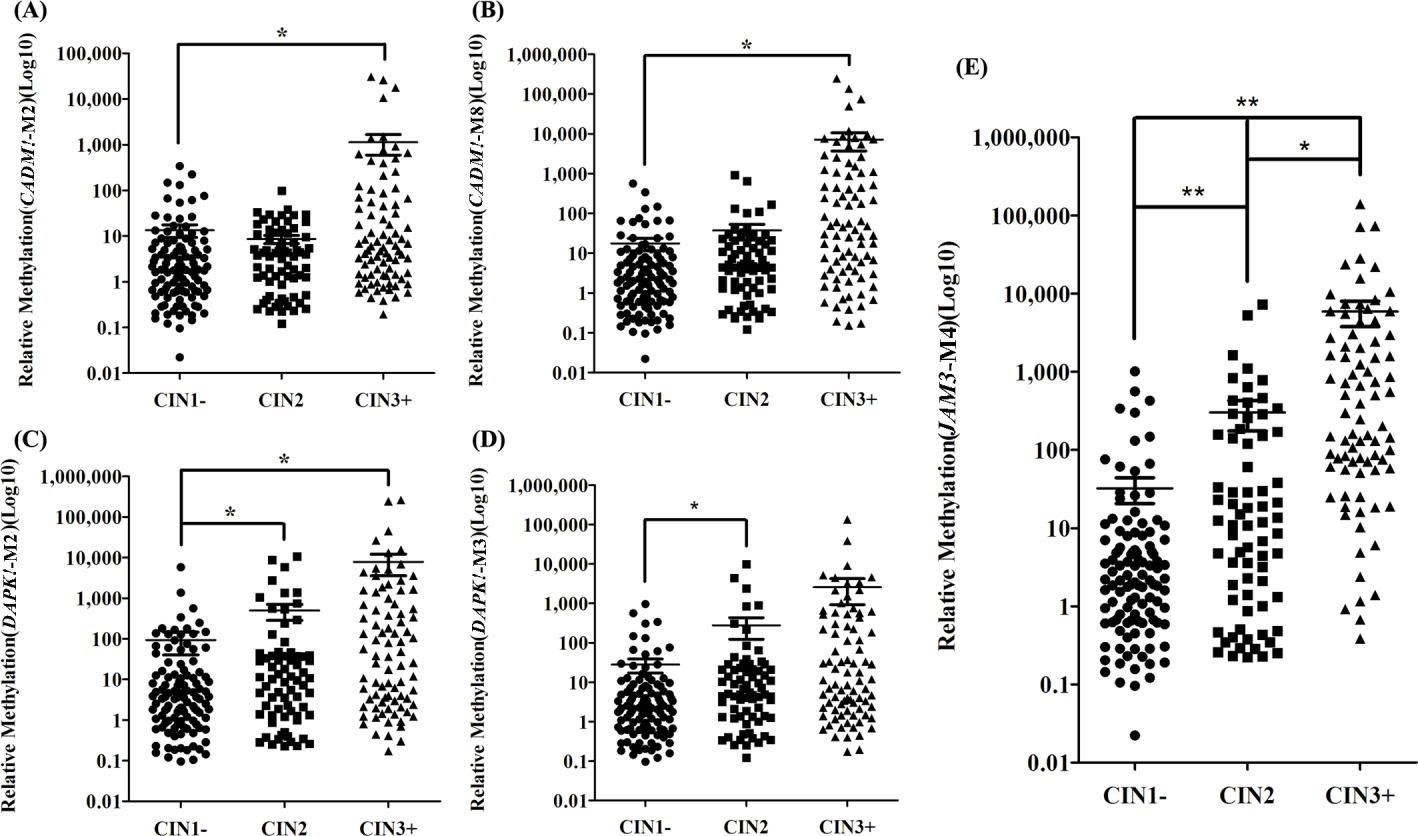
Methylation ratio in cervical scrapings from patients referred for colposcopy (n = 267) Dot plots illustrate the methylation ratio distributions. Methylation ratio of (**A**) *CADM1*-M2, (**B**) *CADM1*-M8, (**C**) *DAPK1*-M2, (**D**) *DAPK1*-M3, (**E**) *JAM3*-M4. CIN1−: no neoplasia and CIN1. CIN3+: CIN3 and cancer cases. *P < 0.05, **P < 0.01. Each point represents one sample; the horizontal line is the mean and whiskers are SEM.

QMSP for *CADM1*-M2, *CADM1*-M8, *DAPK1*-M2, *DAPK1*-M3, and *JAM3*-M4 involved cervical scrapings from a colposcopy referral study (P1) of 267 patients. In general, the methylation ratio increased with increasing lesion severity. Particularly, *JAM3*-M4 was most discriminative marker (Figure 2).

**Figure 3:**
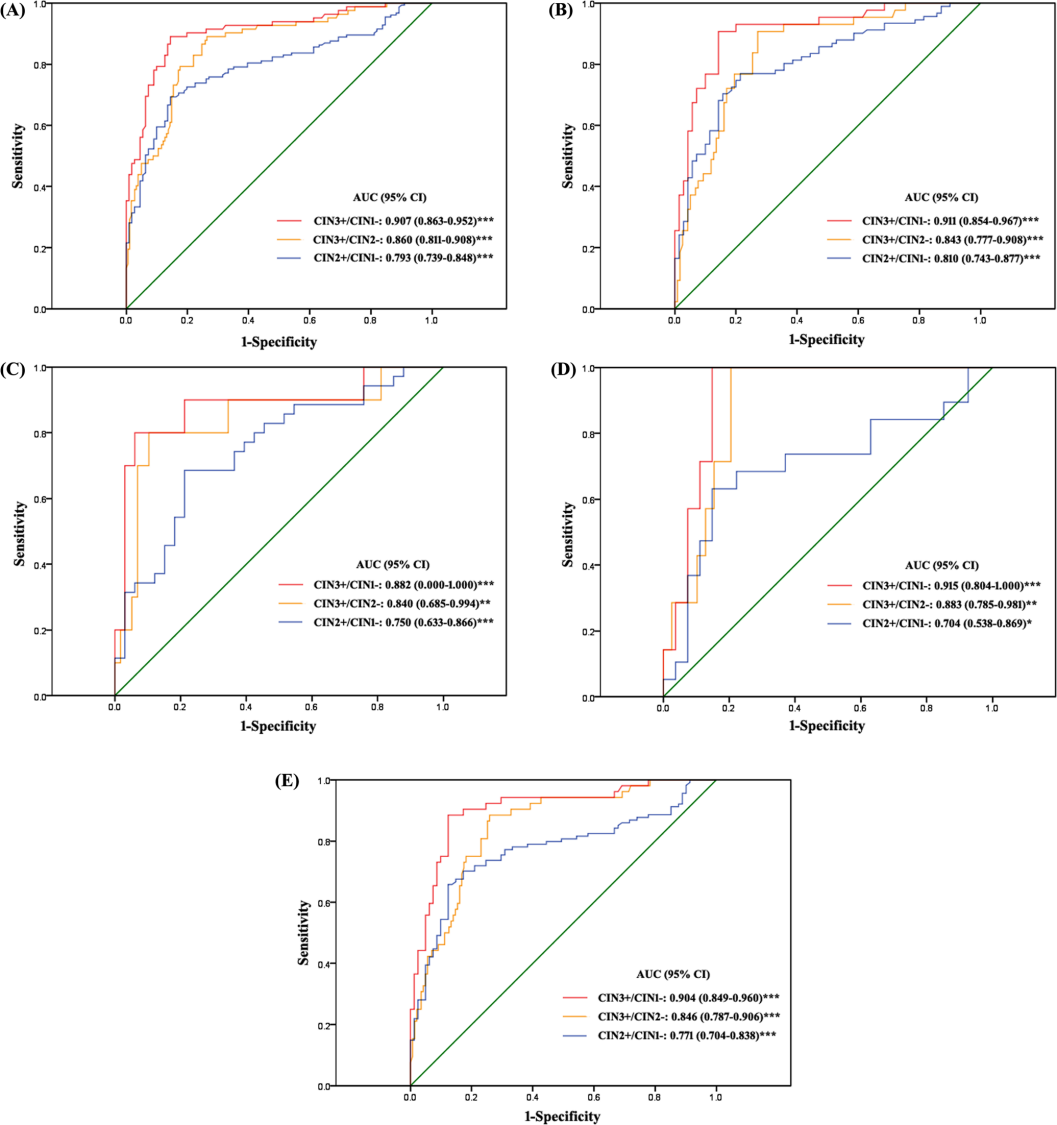
Receiver operating characteristic (ROC) curves of the discriminating performance of JAM3-M4 in different classifications of diagnostic groups in P1 The area under the ROC curve (AUC) was used to estimate accuracy. *JAM3*-M4 performance for (**A**) all patients, (**B**) patients with abnormal cytology result, (**C**) patients with atypical squamous cells of unknown significance (ASCUS), (**D**) patients with low-grade squamous intraepithelial lesion (LSIL), (**E**) patients positive for high-risk human papillomavirus (hrHPV-positive). *P < 0.05, **P < 0.01, ***P < 0.001; 95% CI, 95% confidence interval.

**Figure 4:**
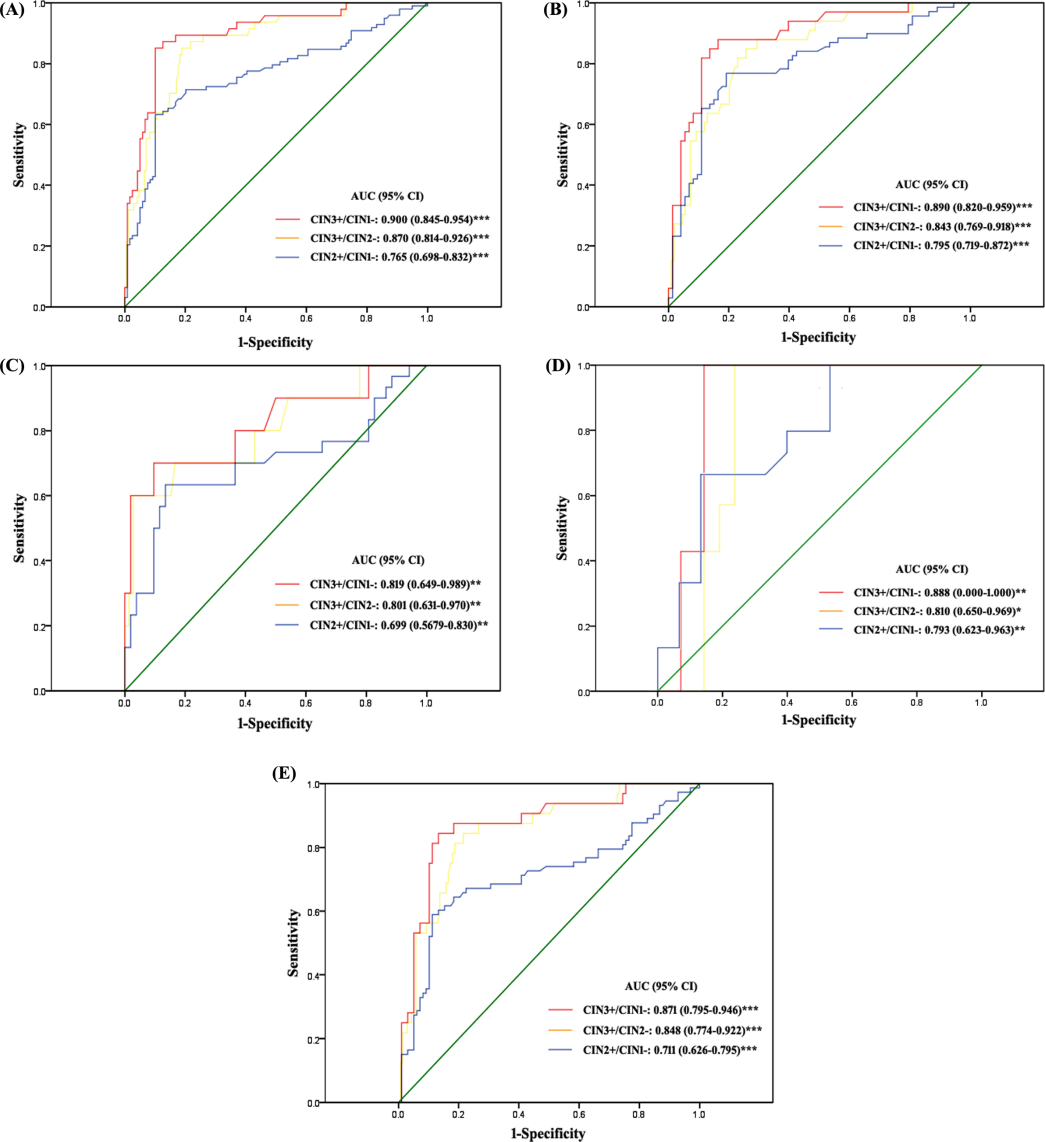
ROC curves of the discriminating performance of JAM3-M4 in different classifications of diagnostic groups in P2 The area under the ROC curve (AUC) was used to estimate accuracy. *JAM3*-M4 performance for (**A**) all patients, (**B**) patients with abnormal cytology result, (**C**) patients with atypical squamous cells of unknown significance (ASCUS), (**D**) patients with low-grade squamous intraepithelial lesion (LSIL), (**E**) patients positive for high-risk human papillomavirus (hrHPV-positive). *P < 0.05, **P < 0.01, ***P < 0.001; 95% CI, 95% confidence interval.

To further investigate the data, we used different classifications for diagnostic groups. CIN2+/CIN1− and CIN3+/CIN2− were the classifications when the end point was CIN2 and CIN3, respectively. Because of the controversy with CIN2 itself, we evaluated the classification CIN3+/CIN1−. A logistic regression model was used to explore the predictive power of methylation of the 5 loci with different diagnostic classifications. For the CIN3+/CIN1− and CIN3+/CIN2− classifications, *JAM3*-M4 had adequate predictive power. Other genes did not add substantial information for discrimination. For the CIN2+/CIN1− classification, *CADM1*-M8 (*P* = 0.001), *DAPK1*-M3 (*P* = 0.038) and *JAM3*-M4 (*P* = 0.011) showed the best discriminating power. However, the area under the receiver operating characteristic curve (AUC) for the 3 genes combined (AUC = 0.806) was only slightly higher than that with *JAM3*-M4 alone (AUC = 0.793). Therefore, in the following analysis, we evaluated *JAM3*-M4 alone with the diagnostic groups in P1 (Figure 3A, Supplementary Table 1). The AUC for *JAM3*-M4 for CIN3+/CIN1−, CIN3+/CIN2− and CIN2+/CIN1− classifications was 0.907, 0.860 and 0.793, respectively. The AUC for *JAM3*-M4 was reproducible in the two studies of P1 and P2, with similar AUC of 0.900, 0.870 and 0.765 for CIN3+/CIN1−, CIN3+/CIN2− and CIN2+/CIN1− classifications in P2 (Figure 4A).

### QMSP of *JAM3*-M4 as a triage marker in patients with abnormal cytology smear results and hrHPV-positive patients

With *JAM3*-M4 used as a triage marker for patients with abnormal cytology smear results (the cutoff was ≥ atypical squamous cells of unknown significance [ASCUS]), the AUC values were 0.911, 0.843 and 0.810 and 0.890, 0.840 and 0.795 for CIN3+/CIN1−, CIN3+/CIN2− and CIN2+/CIN1− classifications in P1 and P2, respectively (Figures 3B, 4B). The sensitivity was slightly decreased and the specificity increased as compared with the triage performance of hrHPV, especially for the CIN3+/CIN1− and CIN3+/CIN2− classifications (Supplementary Table 2). The positive predictive value (PPV) and negative predictive value (NPV) were both increased for all classifications (Supplementary Table 2).

We divided patients with abnormal cytology results into ASCUS and low-grade squamous intraepithelial lesion (LSIL) subgroups and compared the diagnostic performance of *JAM3*-M4 to the triage performance of hrHPV testing (Figures 3C, 4C, 3D, 4D, Tables 4 and 5). For both ASCUS and LSIL subgroups, the specificity and PPV of *JAM3*-M4 was increased significantly for all classification groups, at the cost of a moderate decrease in sensitivity and NPV for ASCUS patients (Tables 4 and 5). Patients with ASCUS who were < 30 years old (*n* = 19) were all hrHPV-positive; 15 had CIN1−, all negative for the *JAM3*-M4 marker.

**Table 4:** Triage performance of methylation marker for patients with hrHPV positive and patients with cytology result of ASCUS and LSIL in P1

	CIN3+/CIN1−				CIN3+/CIN2−				CIN2+/CIN1−			
	SEN (%)	SPE (%)	PPV (%)	NPV (%)	SEN (%)	SPE (%)	PPV (%)	NPV (%)	SEN (%)	SPE (%)	PPV (%)	NPV (%)
ASCUS →HPV	100.00	21.88	26.47	100.00	100.00	12.50	15.52	100.00	100.00	21.88	56.90	100.00
ASCUS →*JAM3*-M4	72.73	81.82	57.14	90.00	72.73	70.69	32.00	93.18	52.78	81.82	76.00	61.36
LSIL →HPV	100.00	15.38	18.52	100.00	100.00	10.81	13.16	100.00	100.00	15.38	42.10	100.00
LSIL →*JAM3*-M4	100.00	85.18	63.64	100.00	100.00	80.00	46.67	100.00	63.16	85.18	75.00	76.67
HPV(+) →Cytology	91.67	30.67	38.82	88.46	91.67	25.58	25.58	91.67	85.56	30.67	59.69	63.89
HPV(+) →*JAM3*-M4	86.79	87.65	82.14	91.02	86.79	73.43	54.76	93.75	65.22	87.65	88.24	63.96

**Table 5:** Triage performance of methylation marker for patients with hrHPV positive and patients with cytology result of ASCUS and LSIL in P2

	CIN3+/CIN1−				CIN3+/CIN2−				CIN2+/CIN1−			
	SEN (%)	SPE (%)	PPV (%)	NPV (%)	SEN (%)	SPE (%)	PPV (%)	NPV (%)	SEN (%)	SPE (%)	PPV (%)	NPV (%)
ASCUS →HPV	100.00	11.36	18.75	100.00	100.00	7.81	13.24	100.00	100.00	11.36	42.65	100.00
ASCUS →*JAM3*-M4	70.00	90.38	58.33	94.00	70.00	81.94	35.00	95.16	50.00	90.38	75.00	75.81
LSIL →HPV*	100.00	0.00	28.57	-	100.00	0.00	25.00	-	100.00	0.00	37.50	-
LSIL →*JAM3*-M4	100.00	78.57	70.00	100.00	100.00	71.43	53.85	100.00	71.43	78.57	76.92	73.33
HPV(+) →Cytology	78.57	43.96	30.14	86.96	78.57	38.58	22.00	89.09	76.56	43.96	49.00	72.73
HPV(+) →*JAM3*-M4	80.64	89.58	71.43	93.48	80.64	80.29	48.08	94.83	58.33	89.58	80.77	74.14

* All the patients with cytology result of LSIL were hrHPV positive.

With *JAM3*-M4 used as a triage marker for hrHPV-positive patients, the AUC was 0.904, 0.846 and 0.771 and 0.871, 0.848 and 0.711 for CIN3+/CIN1−, CIN3+/CIN2− and CIN2+/CIN1− classifications in P1 and P2, respectively (Figures 3E, 4E). *JAM3*-M4 showed slightly decreased sensitivity, with increased specificity, PPV, and NPV as compared with triage performance of cytology testing, especially for CIN3+/CIN1− and CIN3+/CIN2− classifications (Tables 4 and 5).

### QMSP of *JAM3*-M4 as a complementary marker in cytology or hrHPV testing

With *JAM3*-M4 used as a complementary marker in hrHPV or cytology testing, the sensitivity was slightly lower and the specificity and PPV was increased greatly as compared with the combination of cytology and hrHPV testing in P1 (Supplementary Table 3). As most likely used in clinical practice, *JAM3*-M4 as a complementary marker in cytology testing was further confirmed in P2 (Supplementary Table 4).

### Pyrosequencing validation of the methylation status of *JAM3*-M4

**Figure 5:**
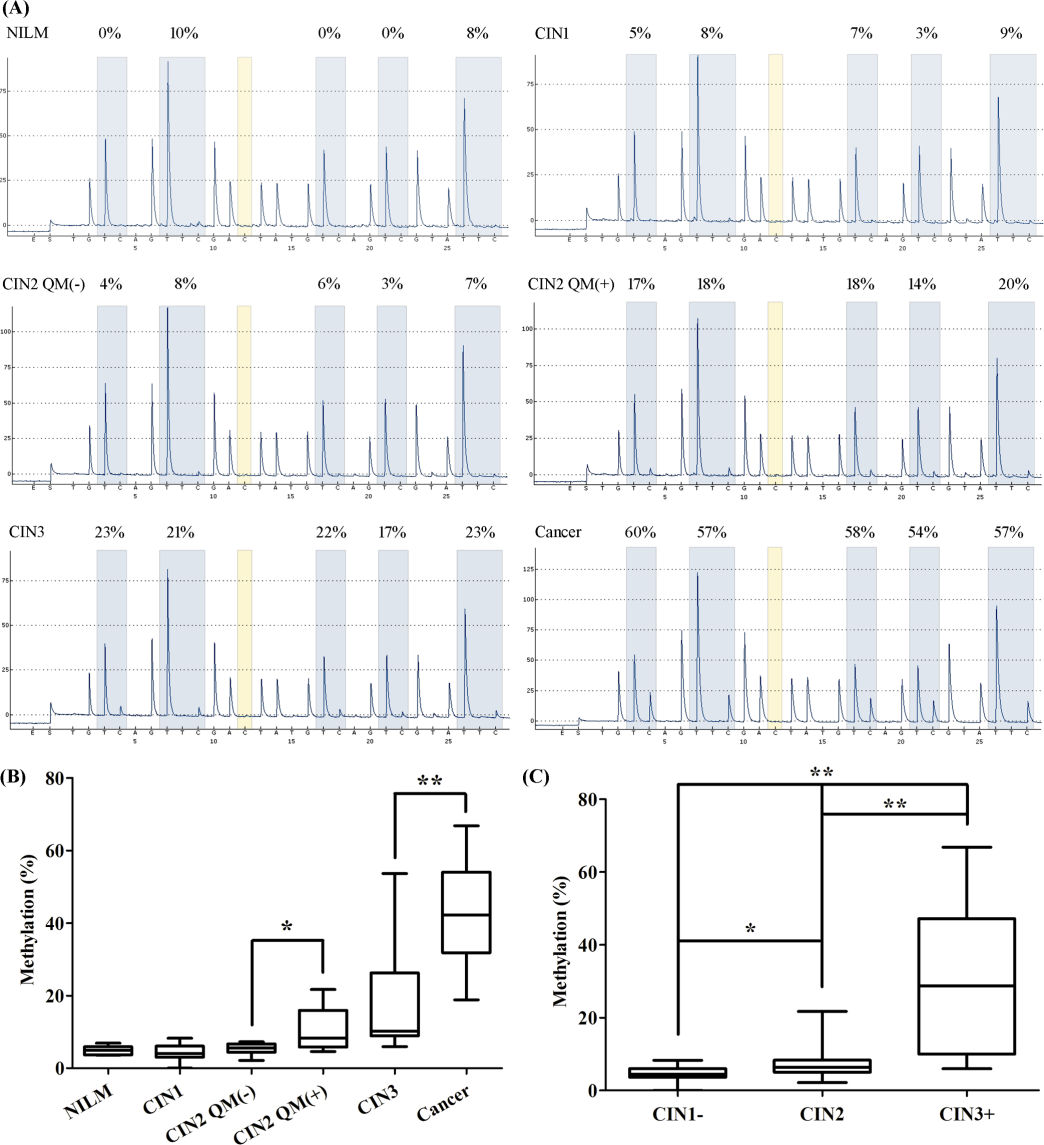
Pyrosequencing analysis of representative loci in JAM3-M4 (**A**) Methylation status of representive loci in representative samples from patient groups: negative for intraepithelial lesion and malignancy (NILM), cervical intraepithelial neoplasia 1 (CIN1), CIN2 QMSP-negative [QM(−)], CIN2 QM(+), CIN3, and cancer confirmed by pyrosequencing. (**B**) Box-plot of the methylation percentage for *JAM3*-M4 for loci for the 6 groups, with statistical analysis between every 2 neighbouring groups. (**C**) Box-plot of the methylation percentage for *JAM3*-M4 for loci for diagnostic groups (CIN1−, CIN2, CIN3+) consistent with the QMSP analysis. Horizontal line is median, whiskers are 5th and 95th percentiles, and lower and upper box boundaries are 25th and 75th percentiles. *P < 0.05, **P < 0.01.

Methylation levels across the CpG sites for *JAM3*-M4 were relatively stable. The mean methylation ratio of the 5 representative CpG sites was 4.94 ± 1.20, 4.36 ± 2.30, 5.37 ± 1.57, 10.68 ± 6.08, 18.49 ± 16.91 and 52.70 ± 14.71 for the patient groups: negative for intraepithelial lesion and malignancy (NILM) (*n* = 8), CIN1 (*n* = 10), CIN2 QMSP-negative (QM(−)) (*n* = 9), CIN2 QM(+) (*n* = 8), CIN3 (*n* = 10) and cancer (*n* = 9) (Figure 5A). The mean methylation ratio of corresponding specimens detected by QMSP and pyrosequencing did not differ (data not shown). Pyrosequencing revealed significant differences between CIN2 QM(−) and CIN2 QM(+), CIN3 and cancer (Figure 5B).

To be consistent with the QMSP analysis of the loci, we examined *JAM3*-M4 discrimination among relevant diagnostic groups (CIN1−, CIN2, CIN3+) and found that it was significantly discriminative (Figure 5C).

### Discriminating performance of *JAM3*-M4 for CIN2 patients

**Figure 6:**
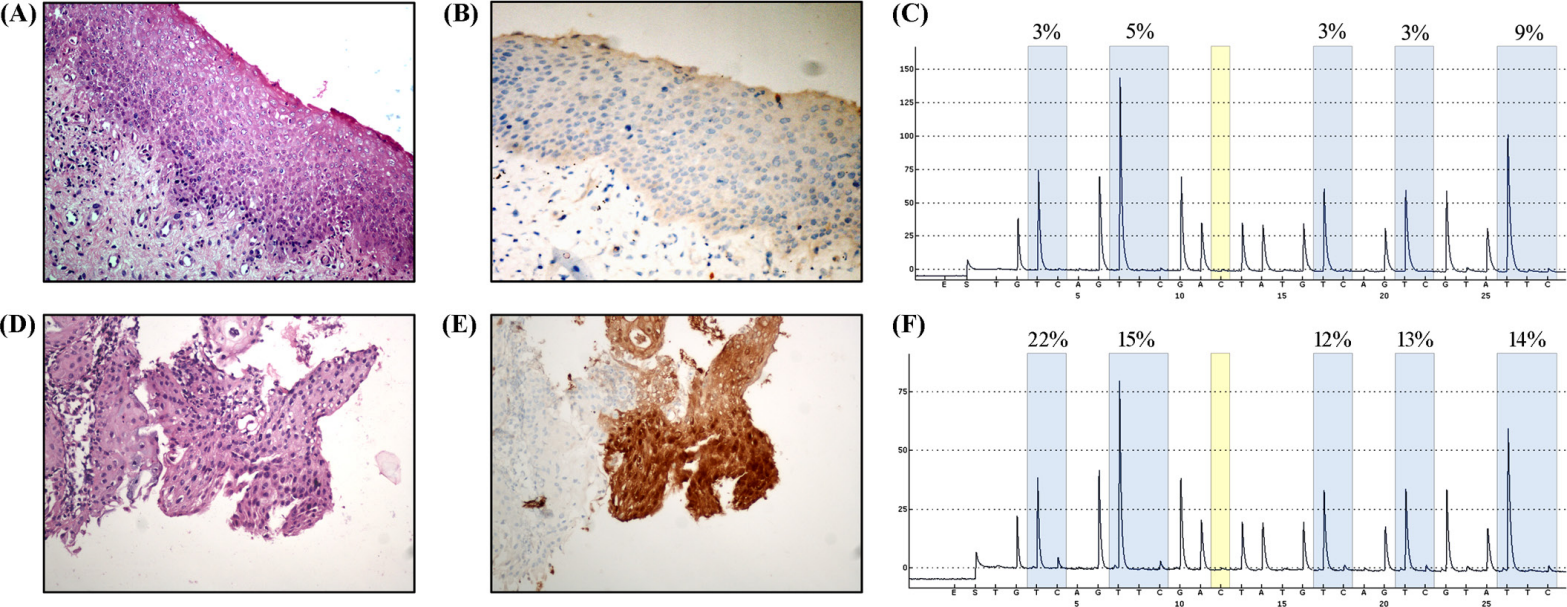
Immunohistochemical staining of P16 in samples from patients with histological diagnosis of CIN2 (**A**) Hematoxylin and eosin staining (H/E) for LSIL, and (**B**) P16, negative in LSIL tissue. (**C**) Pyrosequencing analysis of LSIL. (**D**) H/E staining of HSIL, and (**E**) P16, positive in HSIL tissue. (**F**) Pyrosequencing analysis of HSIL. Original magnification, A, B, C, D, × 200.

Immunohistochemistry analysis of P16 and the corresponding pyrosequencing analysis in samples from representative CIN2 patients are in Figure 6. The rate of positive staining for P16 was 69.9% and the rate of positive methylation for *JAM3*-M4 was 48.8%. The methylation ratio for *JAM3*-M4 between LSIL and HSIL was significant (*P* = 0.03). The coincidence rate was 60.5% (*P* = 0.119).

## DISCUSSION

We used both cervical tissue specimens and cervical scrapings for an in-depth analysis of the methylation status of different loci in candidate gene promoters and their possible diagnostic relevance in cervical neoplasia. *JAM3*-M4 showed good performance in two different and independent studies (P1 and P2) but also with three different diagnostic classifications. *JAM3*-M4 methylation may be a marker for triage and a complementary marker in clinical practice.

Detecting and identifying cervical preneoplasia is important to halt the progression to cancer. As a promising methylation marker, *JAM3* has been investigated for discriminating performance [12, 13, 30, 31]. However, the studies investigated the same locus in different media, including conventional liquid-based cytology, self-sampled brush material and cervico-vaginal lavage. We compared several loci of the same gene and found a new locus, *JAM3*-M4, with promising predictive power in primary screening of cervical neoplasia. We also investigated its combined application with the most widely used screening methods – hrHPV and cytology-based Pap smear testing.

Infection with HPV causes cervical preneoplasia and neoplasia [32]. Depending on the hrHPV testing, the diagnosis can cause over-diagnosis and over-treatment. Unnecessary referral to the gynecologist leads to anxiety, distress [33], and even anger and resentment with a perceived threat to life and/or fertility [34]. Compared to the widely used triage test–cytology after hrHPV testing –*JAM3*-M4 showed increased specificity and PPV. Thus, most unnecessary referrals for colposcopy could be avoided. In addition, with the acceptable PPV of *JAM3*-M4, hrHPV-positive patients also positive for *JAM3*-M4 could be directly referred to colposcopy.

Considering cost-effectiveness, long-term application and high specificity for serious lesions, cytomorphological appraisal is still widely used as primary screening. Developing countries lack quality-controlled cytology testing, and the diagnostic performance is unsatisfactory. The addition of a methylation marker could improve the predictive power even better than combined with HPV testing. Considering the low sensitivity of cytology testing in population-based screening, use of *JAM3*-M4 could be complementary to cytology testing to improve performance, especially the specificity and PPV.

Patients with ASCUS or LSIL account for a considerable proportion of biopsy-confirmed CIN2+ cases [35]. However, the underlying risk of ASCUS or LSIL progressing to CIN2+ is still quite low, from 4% to 8% for ASCUS and 12% to 15% for LSIL [36]. The most widely adopted triage strategy for ASCUS is hrHPV testing. For young patients (< 30 years old), because of the high prevalence of HPV, the test is less effective [37]. As well, because of the high HPV infection rates of LSIL patients – 77% [38] to 80% ~ 85% [39] – such patients need alternative triage methods.

**Figure 7:**
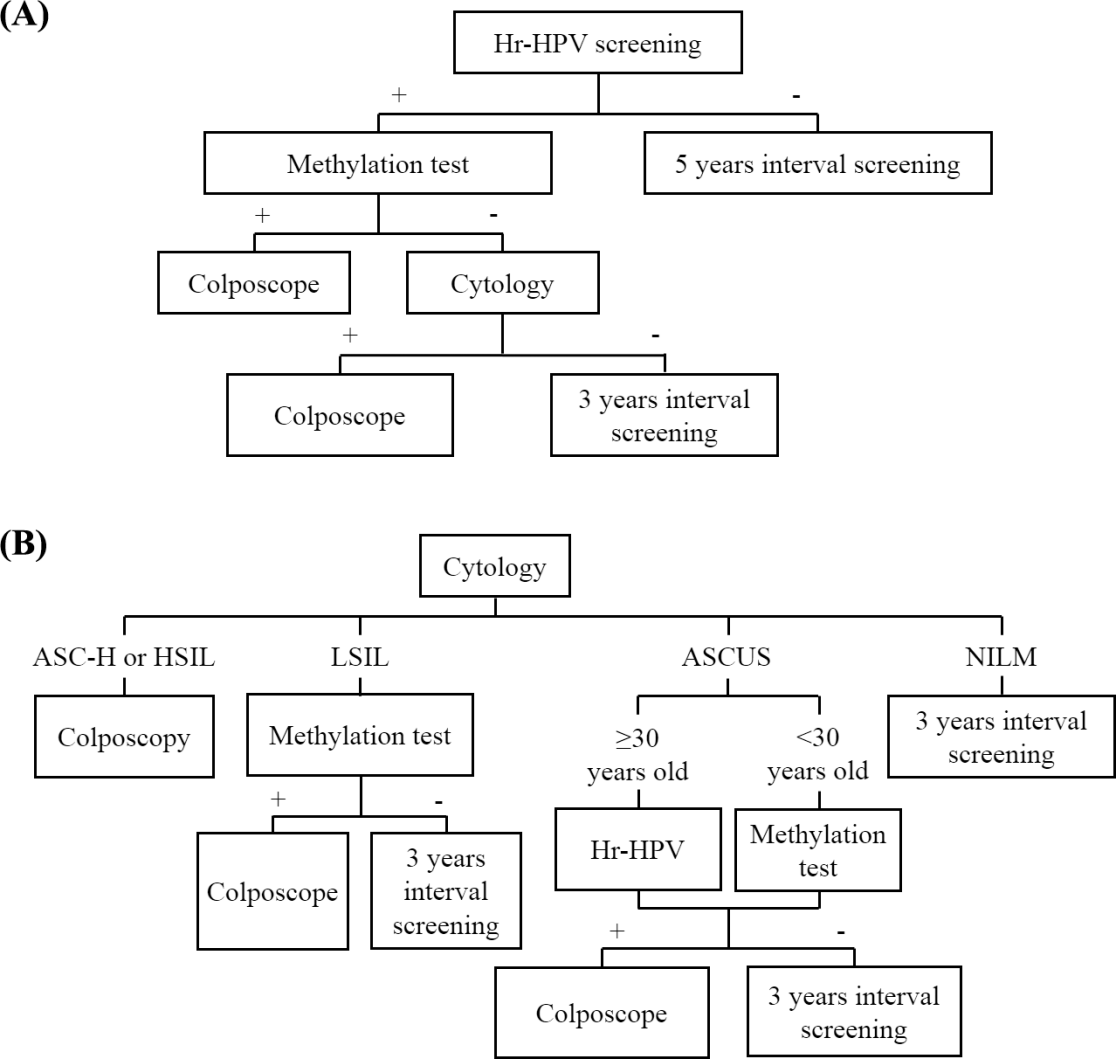
Possible scenarios for the incorporation of a methylation test with JAM3-M4 in cervical cancer screening as a triage marker in (A) HrHPV testing or (B) cytology testing

We investigated the potential of using *JAM3*-M4 in triage of patients with ASCUS and LSIL. Both the specificity and PPV were increased as compared with hrHPV. *JAM3*-M4 could be especially used as a triage marker for patients < 30 years old in light of the less effective triage performance of hrHPV testing in such patients. For LSIL patients, *JAM3*-M4 is ideal as a triage marker without loss of sensitivity and NPV. A proposed scheme for application of this methylation marker in triage is in Figure 7.

CIN lesions are divided into productive (CIN1 and CIN2) and transforming (CIN2 and CIN3) lesions. Productive CIN2 cannot be distinguished from transforming CIN2 by morphology assessment alone [23]. In our study, *JAM3*-M4 performed better with the CIN3+/CIN1− than CIN3+/CIN2− classification. Therefore, the methylation status of CIN2 is also mixed. At present, we lack a well-recognized gold standard to distinguish mixed CIN2. The updated guidelines [40] from the World Health Organization recommend immunohistochemistry analysis of P16 in biopsies to distinguish CIN2 as LSIL or HSIL. Therefore, we compared *JAM3*-M4 methylation status with P16 staining in CIN2 patients. Although results were not statistically significant, *JAM3*-M4 methylation status was still helpful for differentiating LSIL and HSIL.

Because the *JAM3* methylation marker we identified is specific to cervical cancer and discriminative among all diagnostic groups, the role of this gene in carcinogenesis is of interest. JAMs have been described as major components of tight junctions pivotal for establishing and maintaining cell polarity in endothelial and epithelial cells [41, 42]. During tumor development, they are remodeled, thereby allowing neoplastic cells to escape from constraints imposed by intercellular junctions and activate the cytoskeleton machinery into a pro-migratory state of the cell. Overexpression of *JAM3* in an epithelial carcinoma cell line improved tight junctions and restored an epithelial phenotype [43], and the expression was downregulated in gastric adenocarcinoma tissue [44]. *JAM3* promoted hematogenous lung metastasis in melanoma [45] and in an experimental metastatic model *in vivo* [46]. Therefore, its expression and participation vary in tumor cell–endothelial cell interactions in different tumor cells and the specific role in cervical carcinogenesis has not been studied. Investigating the role of *JAM3* in cervical carcinogenesis and whether and which role methylation may play in it would be of interest.

Our study contains some limitations. We examined methylation status in cervical scrapings from patients with a biopsy due to colposcopic abnormalities to avoid verification bias. This cohort was highly selected and was not representative of a screening population. Therefore, this marker may perform differently in a general population of asymptomatic women. Besides squamous cell carcinoma, other clinically important histological variants of cervical cancer, such as adenocarcinoma, exist. We have collected some but not many cervical scrapings of reactive or dysplastic glandular lesion in the cervix. However, *JAM3*-M4 will be further investigated if enough samples are available and may be found a biomarker.

Our study suggests that the *JAM3*-M4 methylation marker may be used as a triage marker for hrHPV-positive patients. For cytology testing, it is also an objective complementary marker, and its detection can be an effective triage strategy for patients with ASCUS, especially those who are < 30 years old and those with LSIL. The performance of this marker should be further evaluated in a prospective, population-based study.

## MATERIALS AND METHODS

### Ethics statement

The investigation was conducted in accordance with the ethical standards and according to the Declaration of Helsinki and national and international guidelines and was approved by the authors' institutional review board.

### Patients

Frozen tissue specimens from 43 cervical cancer and 27 normal cervix were obtained from Qilu Hospital, Shandong University, from January to November 2013. Normal cervix tissue samples were obtained from patients without a history of abnormal cytology smears who planned to undergo hysterectomy for nonmalignant reasons, including fibroids, prolaps uteri, adenomyosis, hypermenorrhea or a combination of these. All cervical tissue was confirmed to be histopathologically normal.

QMSP analysis involved cervical scrapings taken under colposcopic guidance from February 2014 to January 2015 (P1) and July to September 2015 (P2). Samples were selected by random. The sample size was determined with respect to statistical calculation and feasibility and estimated on the basis of significance α, power 1-β and data from preliminary experiments. Only tissue from patients with confirmed histological diagnosis was included. The exclusion criteria were patients with a history of cervical neoplasia, cervix surgery, genital warts, an immunocompromised state, the presence of other cancers, or pregnancy. Histological diagnosis was assessed by 2 qualified pathologists in a blinded fashion. Informed consent was obtained from all patients and controls participating in this study. This study was approved by the ethics committee of Qilu Hospital, Shandong University.

### Cytology testing and hrHPV testing

Cervical smears were cytomorphologically assessed in Thinprep Preservcyt medium (Hologic Inc, USA) by 2 qualified pathologists in a blinded fashion according to the Bethesda nomenclature [47].

Infection with hrHPV was detected by using HC2 kits (Qiagen GmbH, Germany). Samples with a relative light unit (RLU) ratio > 1.0 were recorded as positive.

### DNA extraction and bisulfite modification

Genomic DNA of frozen tissue and cervical scrapings was extracted by using the QlAamp DNA Mini Kit (Qiagen GmbH, Germany). Sodium bisulfate treatment of extracted genomic DNA involved use of EpiTect Bisulfite kits (Qiagen GmbH, Germany). The extracted DNA and modified DNA underwent PCR with primers for the house-keeping gene *GAPDH* (forward: AGGTCGGAGTCAACGGATTTG, reverse: GTGATGGCATGGACTGTGGT) and *β-actin* (forward: TGGTGATGGAGGAGGTTTAGTAAGT, reverse: AACCAATAAAACCTACTCCTCCCTTAA).

### Methylation-sensitive PCR (MSP) and quantitative MSP (QMSP)

MSP was performed on modified genomic DNA. Each PCR was performed in a final volume of 20 μL containing 5 μM each primer, 1 μL bisulfite-conversion DNA, and 1 × AmpliTaq Gold 360 Master Mix (ABI, USA). A sample was considered methylation-positive when a PCR product of the right size was visible after 40 cycles of PCR. The primers are in Table 6.

**Table 6:** MSP and QMSP primers

Gene (locus)	Forward 5′–3′	Reverse 5′–3′	Fragment size (bp)	Tm (°C)
*CADM1*-M2	GTTTTTCGTTATTTGTTGTTTTC	GAAACCGCGAAATACGAACG	117	59
*CADM1*-U2	GTTTTTTGTTATTTGTTGTTTTTG	AAAACCACAAAATACAAACA	117	59
*CADM1*-M8	GTCGTCGTATATTGGGATTC	TCTCATTAACTATCCGCTCG	110	58
*CADM1*-U8	GTTGTTGTATATTGGGATTT	TCTCATTAACTATCCACTCA	110	58
*DAPK1*-M2	CGTTTGTAGGGTTTTTATTGGTC	CTACCGCTACGAATTACCGA	161	60
*DAPK1*-U2	TGTTTGTAGGGTTTTTATTGGTTG	CCTACCACTACAAATTACCA	162	60
*DAPK1*-M3	GTTTTTATTGGTCGTTTGTC	GACGTTAACTCGATCCGACT	102	58
*DAPK1*-U3	TTTTATTGGTTGTTTGTTGG	CCCAACATTAACTCAATCCA	103	58
*JAM3*-M4	CGTAGTTAGGGTTGGGATTC	GAAATCCGACGACTATCCGA	138	60
*JAM3*-U4	TGTAGTTAGGGTTGGGATTT	CAAAATCCAACAACTATCCA	139	60

Abbreviations: Tm: Annealing temperature

QMSP involved 95°C for 10 min, followed by 40 cycles at 95°C for 15 s, 58–60°C for 1 min in a total volume of 20 μL based on the 7900HT Fast Real-Time PCR System (ABI, USA). The primers were identical to those for M markers used in MSP to evaluate the same locus of one gene. The final reaction mixture contained 50 nM each primer, 1 × Power SYBR Green PCR Master Mix (ABI, USA), and 1 μL bisulfite-converted genomic DNA.

EpiTect Control DNA and Control DNA Sets (QIAGEN, Germany; containing both bisulfite-converted methylated and unmethylated DNA, and unconverted unmethylated DNA) were used as MSP and QMSP control DNA. In addition, PCR of the bisulfite converted housekeeping gene β-actin was performed as a reference.

Each sample was analyzed in triplicate. Cycle threshold (Ct) ratios between the Ct values of the β-actin and target were used to quantify the level of methylation, calculated as 2^[Ct (β-actin) - Ct (target)]^ × 10,000. The cut-off value of QMSP for positivity/negativity was calculated and confirmed by receiver operating characteristic (ROC) analysis. Samples with Ct values for β-actin > 32 were considered invalid and excluded from the analysis because they indicated poor DNA quality or recovery after bisulfite treatment.

### Pyrosequencing

Randomly selected samples of different groups underwent pyrosequencing to detect representative loci of *JAM3*-M4 with use of the PyroMark MD system (Qiagen GmbH, Germany) and the frequency of CpG methylation was measured by using PyroMark CpG software.

### Immunohistochemistry

Sections (cervix scrapings and biopsy both collected during colposcopy) from patients histologically confirmed to have CIN2 were used to detect the expression of P16 (Dako, Denmark). Immunostaining involved use of the EnVision detection system (Dako, Denmark) according to the manufacturer's protocol.

### Statistical analysis

Differences in detection rates between cervical cancer and normal cervical specimens by methylation markers were analyzed by chi-square or Fisher's exact test. ROC curves and their cut-off values were determined according to relative methylation levels obtained with QMSP. Pyrosequencing results were analyzed by Mann-Whitney test. Diagnostic performance for triage for methylation markers after hrHPV DNA and cytology testing was expressed as sensitivity, specificity, PPV and NPV with a cut-off for CIN2+ or CIN3+ respectively. All statistical analyses involved use of SPSS 18.0 (SPSS Inc., Chicago, IL, USA). P <0.05 was considered statistically significant.

## SUPPLEMENTARY MATERIAL TABLES


