# Supplementary material for: JAM3 methylation status as a biomarker for diagnosis of preneoplastic and neoplastic lesions of the cervix
Source: Oncotarget. 2015 Oct 27;6(42):44373–87. doi: 10.18632/oncotarget.6250 (PMC4792563; doi:10.18632/oncotarget.6250)
Supplement: Supplementary file 1 [file oncotarget-06-44373-s001.pdf]

JAM3 methylation status as a biomarker for diagnosis of preneoplastic and neoplastic lesions of the cervix

Supplementary Materials

Supplementary Table 1: Diagnostic performance of different methods for distinguishing different diagnostic groups in P1

	CIN3+/CIN1−				CIN3+/CIN2−				CIN2+/CIN1−			
	SEN (%)	SPE (%)	PPV (%)	NPV (%)	SEN (%)	SPE (%)	PPV (%)	NPV (%)	SEN (%)	SPE (%)	PPV (%)	NPV (%)
HPV	98.15	16.33	39.26	94.12	98.15	10.56	26.90	94.44	98.29	16.33	58.38	88.89
Cytology	91.67	27.37	38.94	86.67	91.67	23.22	26.99	90.00	86.79	26.80	56.44	65.00
JAM3-M4	87.95	85.58	82.02	90.48	87.95	73.63	60.33	93.06	68.83	85.58	86.88	66.43

Abbreviations: Sen: sensitivities, Spe: specificities, PPV: positive predictive value, NPV: negative predictive value

Supplementary Table 2: Triage performance of methylation marker for patients with abnormal cytology smear in P1

	CIN3+/CIN1−				CIN3+/CIN2−				CIN2+/CIN1−			
	SEN (%)	SPE (%)	PPV (%)	NPV (%)	SEN (%)	SPE (%)	PPV (%)	NPV (%)	SEN (%)	SPE (%)	PPV (%)	NPV (%)
Cytology (+)→HPV	97.06	1.89	38.82	50.00	97.06	12.73	25.58	93.33	98.72	1.89	59.69	50.00
Cytology (+)→*JAM3*-M4	88.64	84.28	78.00	92.19	88.64	70.34	52.70	94.32	69.56	84.28	85.33	67.82

Supplementary Table 3: Complementary performance of methylation marker for hrHPV testing and cytology testing in P1

	CIN3+/CIN1−				CIN3+/CIN2−				CIN2+/CIN1−			
	SEN (%)	SPE (%)	PPV (%)	NPV (%)	SEN (%)	SPE (%)	PPV (%)	NPV (%)	SEN (%)	SPE (%)	PPV (%)	NPV (%)
HPV + Cytology	89.19	41.57	38.82	90.24	89.19	33.33	25.58	92.31	83.70	41.57	59.69	71.15
HPV + *JAM3*-M4	85.18	89.69	82.14	91.58	85.18	76.25	54.76	93.85	64.10	89.69	88.24	67.44
Cytology + *JAM3-M4*	81.25	88.54	78.00	90.42	81.25	77.27	52.70	92.97	60.38	88.54	85.33	66.93

Supplementary Table 4: Complementary performance of methylation marker for cytology testing in P2

	CIN3+/CIN1−				CIN3+/CIN2−				CIN2+/CIN1−			
	SEN (%)	SPE (%)	PPV (%)	NPV (%)	SEN (%)	SPE (%)	PPV (%)	NPV (%)	SEN (%)	SPE (%)	PPV (%)	NPV (%)
Cytology + HPV	75.86	47.42	30.14	86.79	75.86	41.35	22.00	88.71	75.38	47.42	49.00	74.19
Cytology + *JAM3*-M4	67.50	92.04	75.00	88.89	67.50	82.91	50.00	90.97	52.94	92.04	83.33	72.22
